# Real-world effectiveness of palbociclib plus endocrine therapy in HR+/HER2− advanced breast cancer: final results from the POLARIS trial

**DOI:** 10.1093/oncolo/oyae291

**Published:** 2024-10-30

**Authors:** Debu Tripathy, Joanne L Blum, Meghan S Karuturi, Steven McCune, Sobha Kurian, Mehdi Moezi, Daniel Anderson, Eric Gauthier, Zhe Zhang, Monica Z Montelongo, Yao Wang, Gabrielle B Rocque

**Affiliations:** Department of Breast Medical Oncology, The University of Texas MD Anderson Cancer Center, Houston, TX 77030, United States; Baylor-Sammons Cancer Center, Texas Oncology, US Oncology, Dallas, TX 75246, United States; Department of Breast Medical Oncology, The University of Texas MD Anderson Cancer Center, Houston, TX 77030, United States; Wellstar Health System, Marietta, GA 30060, United States; West Virginia University Cancer Institute, Morgantown, WV 26506, United States; Cancer Specialists of North Florida, Fleming Island, FL 32003, United States; Metro-Minnesota Community Oncology Research Consortium, Health Partners Institute, St. Paul, MN 55425, United States; Pfizer Inc., San Francisco, CA 94105, United States; Pfizer Inc., La Jolla, CA 92121, United States; ICON plc, Blue Bell, PA 19422, United States; Pfizer Inc., La Jolla, CA 92121, United States; Hematology and Oncology, University of Alabama at Birmingham, Birmingham, AL 35233, United States

**Keywords:** palbociclib, real-world, advanced breast cancer, HR+/HER2−

## Abstract

**Background:**

Strict eligibility criteria for participation in randomized clinical trials (RCTs) often limit the generalizability of data when applied to a more heterogeneous real-world population. Thus, evidence generated directly from real-world populations, including subgroups underrepresented in RCTs, can help inform routine clinical practice. POLARIS (NCT03280303), a prospective, observational, multicenter, cohort study, evaluated patients with hormone receptor-positive/human epidermal growth factor receptor 2-negative (HR+/HER2−) advanced breast cancer (ABC) receiving palbociclib + endocrine therapy (ET) in routine care.

**Methods:**

Demographics, baseline characteristics, and treatment patterns were summarized descriptively. Real-world response and clinical benefit rates, real-world progression-free survival (rwPFS), and overall survival (OS) were summarized descriptively by line of therapy and endocrine partner in the overall cohort and various subgroups.

**Results:**

Between January 2017 and October 2019, 1250 patients (median age of 64.0 years) initiated treatment with palbociclib-based therapy, including 901 in the first-line (1L) setting and 349 in the second-line or later (≥2L) settings. Real-world response and clinical benefit rates with palbociclib + ET were 34.0% and 69.4%, respectively, in 1L, and 21.8% and 57.9% in ≥2L. Median rwPFS was 20.9 (95% CI, 18.7-24.7) and 13.5 (10.6-17.1) months, and median OS was 48.5 (42.0-not estimable) and 37.2 (31.2-40.8) months, with 1L and ≥2L palbociclib + ET, respectively.

**Conclusions:**

Outcomes in this large, heterogeneous, real-world population are generally consistent with previously reported results from clinical trials and other real-world studies, further supporting the use of palbociclib + ET in patients with HR+/HER2− ABC.

**Trial registration:**

NCT03280303 (ClinicalTrials.gov).

**Implications for practice**

Strict eligibility criteria in clinical trials often limit the diversity of patients enrolled and, consequently, the generalizability of findings to patients in routine clinical practice. Therefore, robust real-world evidence is needed to further understand treatment effectiveness in the real-world setting. POLARIS, a prospective, multicenter, non-interventional, real-world study, described the effectiveness of palbociclib + endocrine therapy (ET) in a large, heterogeneous (in terms of sex, menopausal status, age, etc.) population of patients with HR+/HER2− advanced breast cancer (ABC). Together with prior clinical trial and real-world data, this study helps support the use of palbociclib + ET for patients with HR+/HER2– ABC.

## Introduction

Hormone receptor-positive/human epidermal growth factor receptor 2-negative (HR+/HER2−) advanced breast cancer (ABC) is generally incurable.^1^ In recent years, large, randomized multicenter trials have demonstrated that the addition of novel targeted therapies to traditional endocrine therapy (ET) could significantly improve the clinical outcome for many of these patients.^1-4^ However, strict eligibility criteria in clinical trials often limit the diversity of patients enrolled by excluding patients based on, for example, select comorbidities, concomitant medication usage, older age, sex, worse performance status, or organ dysfunction, among other factors.^5-9^ Consequently, generalizability of randomized clinical trial (RCT) findings to the real-world setting can be limited.^5^ To fill this clinical gap in evidence, real-world evidence provides an opportunity to understand drug effectiveness in actual practice in a more heterogeneous population of patients with ABC.^10^

The addition of cyclin-dependent kinase 4/6 (CDK4/6) inhibitors to ET has become the standard of care for patients with HR+/HER2− ABC,^1,4,11^ with roughly a doubling of median progression-free survival (PFS) vs ET alone reported across a number of different clinical trials in the first-line (1L), second-line (2L), or later setting.^12-20^ Palbociclib, the first-in-class CDK4/6 inhibitor, is approved for the treatment of adult patients with HR+/HER2− ABC in combination with an aromatase inhibitor (AI) as the initial ET-based regimen, or with fulvestrant in patients with disease progression following ET.^21,22^ The pivotal phase III trials supporting these indications were PALOMA-2 and PALOMA-3, respectively.^22^ Since the introduction of palbociclib, real-world evidence has been generated, adding to the body of evidence beyond RCTs and helping inform decision-making in routine clinical practice.^23-26^ Furthermore, robust long-term data from large real-world studies offer opportunities to improve understanding of palbociclib prescribing patterns and associated clinical outcomes in patients with HR+/HER2*−* ABC who are managed in routine clinical practice, especially for subpopulations that are often underrepresented in breast cancer clinical trials (eg, older, racial/ethnic minority, or male subpopulations).^27-29^

The POLARIS trial (NCT03280303), a longitudinal study of patients with HR+/HER2− ABC receiving treatment with palbociclib in routine clinical practice, was designed to prospectively collect real-world data on palbociclib prescribing and treatment patterns, clinical outcomes, treatment sequencing, patient quality of life, longitudinal tumor biomarker/genomic assessments, and geriatric-specific assessments. Here we report real-world treatment patterns and effectiveness outcomes from POLARIS.

## Methods

### Study design

POLARIS (NCT03280303) is a prospective, observational, multicenter, real-world cohort study conducted in >100 sites in the US and Canada. Detailed methods have been previously published.^30^ Demographic, treatment, quality of life, and disease assessment data, as well as serial blood samples, were collected from routine clinical assessments performed by the treating physicians. Electronic case report forms were used for clinical and treatment data collection. Trained investigators or authorized medical staff recorded data from existing medical records into an electronic case report form following each patient’s standard-of-care clinic visit. OmniComm Systems, Inc., was used as the web-based electronic data capture system to collect, monitor, and report clinical data. Remote data monitors reviewed all data collected via electronic case report forms and queried study sites regarding missing or unclear data to ensure data integrity, consistency, and completeness.

Eligible patients were aged ≥18 years; diagnosed with breast adenocarcinoma with evidence of metastatic disease or advanced disease not amenable to treatment with curative intent; have documented HR+ (estrogen and/or progesterone receptor–positive) and HER2− tumor based on local standards; and considered to be a candidate to receive treatment with palbociclib by their treating physician. Exclusion criteria included a life expectancy of <3 months at the time of ABC diagnosis, per the investigator’s judgment; participation in any interventional clinical trial that included investigational or approved drugs at the time of enrollment; receipt of active treatment for malignancies other than ABC at the time of enrollment; and an inability to provide informed consent. Patients were followed from the start of palbociclib treatment until approximately 3 years after the end of palbociclib treatment, patient withdrawal from the study, or death, whichever came first.

All patients provided written informed consent before participating in the study. The study was conducted in accordance with local regulatory and legal requirements to ensure confidentiality and protection of patients’ personal data. The study was reviewed and approved by applicable local institutional review boards or independent ethics committees.

### Outcomes

In this analysis, line of therapy was defined as the number of systemic therapies received after the initial diagnosis of ABC, but before the start of palbociclib treatment. Patients receiving 1L treatment had no prior lines of therapy in the ABC setting before palbociclib initiation, and patients receiving ≥2L treatment had ≥1 prior lines of therapy in the ABC setting before palbociclib initiation. Disease-free interval (DFI) was defined as the time from date of first diagnosis of primary breast cancer to first onset of relapse/recurrent disease among patients with non-missing dates. Patients with initial breast cancer diagnosis of metastatic breast cancer (MBC; ie, de novo metastatic disease) were excluded from the DFI interval calculation.

Real-world tumor responses (real-world complete response [rwCR], real-world partial response [rwPR], real-world stable disease [rwSD], and real-world progressive disease) during the respective line of therapy (after palbociclib initiation but before the start date of the next line of treatment) were determined by the treating physicians based on imaging, biopsies, biomarkers, and/or clinical judgment in routine clinical practice. Real-world response rate (rwRR) was defined as the proportion of patients with a best response of either rwCR or rwPR in patients with at least 1 disease assessment during the respective line of therapy. Real-world clinical benefit rate (rwCBR) was defined as the proportion of patients with a best response of rwCR or rwPR at any time, or rwSD for at least 24 weeks, in patients with at least 1 disease assessment during the respective line of therapy.

Real-world PFS (rwPFS) was defined as the time (in months) from study treatment initiation until physician-documented disease progression (based on imaging, biopsies, biomarkers, and/or clinical judgment) or death due to any cause, whichever occurred first, during the respective line of therapy. Patients who did not have physician-documented progression or documented death were censored at the last date of response assessment without progressive disease through the start date of the next line of therapy (for patients with ≥1 line of therapy after palbociclib), or their last date of response assessment without progressive disease during the study period (for patients who did not receive a subsequent line of therapy after palbociclib).

Overall survival (OS) was defined as the time from study treatment initiation until death due to any cause. Patients who did not have a documented death were censored at the last available visit date when they were known to be alive.

### Statistical analyses

POLARIS is a prospective, observational study with the sample size estimated to ensure adequate precision of estimates under large amounts of variability and attrition in the population and to allow sufficient generalizability. All analyses were descriptive in nature.

The analysis population consisted of all enrolled patients who received at least 1 dose of palbociclib as of the data cutoff (safety analysis set). Patient demographics and treatment patterns were summarized with descriptive statistics. All available patient data were used for the analysis with missing or unknown categories reported for each variable, where applicable. Effectiveness outcomes (rwRR, rwCBR, rwPFS, and OS) were summarized by line of therapy in which palbociclib was given, and by an endocrine partner. Effectiveness outcomes were also assessed in the per-label population, consisting of patients in the safety analysis set who had HR+/HER2− disease and were treated as per the US label,^22^ defined as palbociclib + AI in the 1L setting or palbociclib + fulvestrant after prior ET in any setting. Medians and associated 95% CIs for rwPFS and OS were estimated using the Kaplan-Meier method. Duration of follow up for rwPFS and OS was calculated using the reverse Kaplan-Meier method, where rwPFS or OS events, respectively, were censored.

Subgroup analyses evaluated effectiveness outcomes by key patient baseline characteristics including age (<65 or ≥65 years), sex (male or female), race (Black, Indigenous, and People of Color [BIPOC] category, yes or no), menopausal status (pre/perimenopausal or postmenopausal), number of disease sites at the time of treatment initiation (1 or ≥2), bone-only disease (yes or no), and visceral disease (yes or no).

## Results

### Patients

Between January 04, 2017 and October 03, 2019, 1285 patients enrolled in POLARIS, of whom 1250 initiated treatment with palbociclib with or without ET as 1L or ≥2L therapy and were included in this analysis; the data cutoff date for this analysis was January 09, 2023. The patient disposition for the overall POLARIS study population is shown in Supplementary Figure S1. The median age was 64 years, and 33.1% of patients were ≥70 years of age. Patients were predominantly White (81.8%), followed by Black/African American (11.1%), and Asian (1.8%; Table 1). At the time of enrollment, 27.3% of patients had de novo stage IV metastatic disease. Of patients with metastatic disease at study enrollment (*n* = 1186), 41.7% and 34.1% had visceral disease or bone-only metastases, respectively, at the time of MBC diagnosis.

**Table 1.** Patient baseline demographic and disease characteristics.

	1L therapy (*n* = 901)			≥2L therapy (*n* = 349)			Overall patients^a^^,^^b^ (*N* = 1250)
	PAL + AI (*n* = 573)	PAL + FUL (*n* = 308)	1L overall^a^ (*n* = 901)	PAL + AI (*n* = 154)	PAL + FUL (*n* = 184)	≥2L overall^b^ (*n* = 349)	
Age							
*n* (missing)	571 (2)	307 (1)	898 (3)	153 (1)	184 (0)	348 (1)	1246 (4)
Median (range), years	64.0 (22-97)	64.0 (24-91)	64.0 (22-97)	63.0 (27-87)	65.0 (36-92)	63.5 (27-92)	64.0 (22-97)
Distribution, *n* (%)							
<50 years	82 (14.4)	43 (14.0)	131 (14.6)	39 (25.5)	21 (11.4)	62 (17.8)	193 (15.5)
≥50-<70 years	308 (53.9)	150 (48.9)	466 (51.9)	69 (45.1)	97 (52.7)	174 (50.0)	640 (51.4)
≥70 years	181 (31.7)	114 (37.1)	301 (33.5)	45 (29.4)	66 (35.9)	112 (32.2)	413 (33.1)
Sex, *n* (%)							
Male	5 (0.9)	4 (1.3)	9 (1.0)	2 (1.3)	4 (2.2)	6 (1.7)	15 (1.2)
Female	568 (99.1)	304 (98.7)	892 (99.0)	152 (98.7)	180 (97.8)	343 (98.3)	1235 (98.8)
Race, *n* (%)							
White	469 (81.8)	254 (82.5)	737 (81.8)	123 (79.9)	153 (83.2)	285 (81.7)	1022 (81.8)
Black/African American	71 (12.4)	25 (8.1)	100 (11.1)	18 (11.7)	20 (10.9)	39 (11.2)	139 (11.1)
Asian	8 (1.4)	7 (2.3)	15 (1.7)	5 (3.2)	3 (1.6)	8 (2.3)	23 (1.8)
American Indian/Alaska Native	3 (0.5)	3 (1.0)	6 (0.7)	0	1 (0.5)	2 (0.6)	8 (0.6)
Native Hawaiian/other Pacific Islander	2 (0.3)	3 (1.0)	5 (0.6)	0	0	0	5 (0.4)
Other	8 (1.4)	6 (1.9)	14 (1.6)	4 (2.6)	4 (2.2)	8 (2.3)	22 (1.8)
Not reported or missing	12 (2.1)	10 (3.2)	24 (2.7)	4 (2.6)	3 (1.6)	7 (2.0)	31 (2.5)
Ethnicity, *n* (%)							
Not Hispanic/Latino	524 (91.4)	258 (83.8)	796 (88.3)	133 (86.4)	168 (91.3)	310 (88.8)	1106 (88.5)
Hispanic/Latino	30 (5.2)	41 (13.3)	76 (8.4)	16 (10.4)	13 (7.1)	30 (8.6)	106 (8.5)
Not reported or missing	19 (3.3)	9 (2.9)	29 (3.2)	5 (3.2)	3 (1.6)	9 (2.6)	38 (3.0)
Menopause status							
*n* (not applicable)	568 (5)	304 (4)	892 (9)	152 (2)	180 (4)	343 (6)	1235 (15)
Distribution, *n* (%)							
Postmenopausal	486 (85.6)	273 (89.8)	776 (87.0)	124 (81.6)	161 (89.4)	294 (85.7)	1070 (86.6)
Pre/perimenopausal	75 (13.2)	29 (9.5)	107 (12.0)	24 (15.8)	15 (8.3)	40 (11.7)	147 (11.9)
Unknown	7 (1.2)	2 (0.7)	9 (1.0)	4 (2.6)	4 (2.2)	9 (2.6)	18 (1.5)
Stage of diagnosis at study enrollment, *n* (%)							
Locally advanced (stage III)	24 (4.2)	17 (5.5)	42 (4.7)	7 (4.5)	11 (6.0)	20 (5.7)	62 (5.0)
Metastatic (stage IV)	548 (95.6)	290 (94.2)	857 (95.1)	147 (95.5)	173 (94.0)	329 (94.3)	1186 (94.9)
Not reported	1 (0.2)	1 (0.3)	2 (0.2)	0	0	0	2 (0.2)
Molecular subtype at most recent recurrence diagnosis prior to enrolment (or initial diagnosis, if no recurrence), *n* (%)							
HR+/HER2−	533 (93.0)	293 (95.1)	846 (93.9)	139 (90.3)	172 (93.5)	320 (91.7)	1166 (93.3)
HR−/HER2−	2 (0.3)	1 (0.3)	3 (0.3)	3 (1.9)	1 (0.5)	4 (1.1)	7 (0.6)
HR+/HER2+	8 (1.4)	6 (1.9)	14 (1.6)	3 (1.9)	3 (1.6)	7 (2.0)	21 (1.7)
HR−/HER2+	0	0	0	0	0	0	0
Unknown	25 (4.4)	7 (2.3)	32 (3.6)	7 (4.5)	7 (3.8)	15 (4.3)	47 (3.8)
Not reported	5 (0.9)	1 (0.3)	6 (0.7)	2 (1.3)	1 (0.5)	3 (0.9)	9 (0.7)
Disposition of diagnosis at study enrolment, *n* (%)							
Recurrent from earlier stage, stages 0-III	352 (61.4)	276 (89.6)	642 (71.3)	93 (60.4)	108 (58.7)	207 (59.3)	849 (67.9)
De novo, newly diagnosed stage IV	186 (32.5)	25 (8.1)	217 (24.1)	56 (36.4)	64 (34.8)	124 (35.5)	341 (27.3)
Not reported	35 (6.1)	7 (2.3)	42 (4.7)	5 (3.2)	12 (6.5)	18 (5.2)	60 (4.8)
Time from ABC/MBC diagnosis to enrolment							
*n* (missing)	570 (3)	307 (1)	897 (4)	154 (0)	184 (0)	349 (0)	1246 (4)
Median (range), months	0.9 (0-248)	0.9 (0-86)	0.9 (0-248)	16.6 (0-242)	31.4 (1-191)	26.5 (0-242)	1.3 (0 − 248)
Distribution, *n* (%)							
≤1 month	322 (56.5)	171 (55.7)	505 (56.3)	7 (4.5)	2 (1.1)	9 (2.6)	514 (41.3)
>1-≤2 months	150 (26.3)	74 (24.1)	230 (25.6)	6 (3.9)	9 (4.9)	15 (4.3)	245 (19.7)
>2-≤6 months	65 (11.4)	27 (8.8)	93 (10.4)	22 (14.3)	11 (6.0)	35 (10.0)	128 (10.3)
>6 months	33 (5.8)	35 (11.4)	69 (7.7)	119 (77.3)	162 (88.0)	290 (83.1)	359 (28.8)
Disease-free interval^c^							
*n* (missing)	384 (3)	282 (1)	680 (4)	98 (0)	120 (0)	225 (0)	905 (4)
Median (range), months	85.9 (0-435)	63.8 (0-424)	74.7 (0-435)	78.3 (0-365)	77.7 (0-410)	79.2 (0-410)	75.2 (0-435)
Distribution, *n* (%)							
No disease-free interval^d^	8 (2.1)	4 (1.4)	12 (1.8)	1 (1.0)	7 (5.8)	9 (4.0)	21 (2.3)
≤12 months	55 (14.3)	13 (4.6)	70 (10.3)	14 (14.3)	13 (10.8)	28 (12.4)	98 (10.8)
>12-≤24 months	21 (5.5)	14 (5.0)	37 (5.4)	5 (5.1)	5 (4.2)	10 (4.4)	47 (5.2)
>24-≤36 months	23 (6.0)	42 (14.9)	67 (9.9)	12 (12.2)	9 (7.5)	21 (9.3)	88 (9.7)
>36 months	277 (72.1)	209 (74.1)	494 (72.6)	66 (67.3)	86 (71.7)	157 (69.8)	651 (71.9)
Sites of distant metastases at MBC diagnosis^e^							
*n* (missing)	548 (0)	290 (0)	857 (0)	146 (1)	173 (0)	328 (1)	1185 (1)
Median (range) number of sites	1.0 (1-10)	2.0 (1-10)	1.0 (1-10)	2.0 (1-9)	2.0 (1-7)	2.0 (1-9)	2.0 (1-10)
Bone involvement at MBC diagnosis^e^, *n* (%)							
Bone + other metastases	205 (37.4)	107 (36.9)	318 (37.1)	63 (42.9)	95 (54.9)	163 (49.5)	481 (40.6)
Bone-only	213 (38.9)	97 (33.4)	319 (37.2)	42 (28.6)	44 (25.4)	86 (26.1)	405 (34.1)
Visceral disease^f^ at MBC diagnosis^e^, n (%)							
Yes	192 (35.0)	130 (44.8)	328 (38.3)	72 (49.0)	86 (49.7)	166 (50.5)	494 (41.7)
No	356 (65.0)	160 (55.2)	529 (61.7)	75 (51.0)	87 (50.3)	163 (49.5)	692 (58.3)

^a^Includes data from 20 patients who received 1L treatment with other palbociclib regimens (defined as palbociclib with no endocrine partners, or palbociclib with endocrine partners other than letrozole, anastrozole, fulvestrant, or exemestane), which are reported in Supplementary Table S1.

^b^Includes data from 11 patients who received ≥2L treatment with other palbociclib regimens (defined as palbociclib with no endocrine partners, or palbociclib with endocrine partners other than letrozole, anastrozole, fulvestrant, or exemestane), which are reported in Supplementary Table S1.

^c^Disease-free interval from first diagnosis of breast cancer to first onset of relapse/recurrent disease among patients with non-missing dates. Patients with initial breast cancer diagnosis of MBC (ie, de novo metastatic disease) are excluded from the disease-free interval calculation.

^d^No disease-free interval indicates that the initial diagnosis date is the same as ABC/MBC diagnosis date in patients with non-MBC initial diagnosis.

^e^Among patients with metastatic disease at study enrollment.

^f^Metastases of the brain, liver, and/or lung/pleura.

Abbreviations: 1L, first-line; 2L, second-line; ABC, advanced breast cancer; AI, aromatase inhibitor; FUL, fulvestrant; MBC, metastatic breast cancer; PAL, palbociclib.

### Treatment patterns

**Figure 1.**
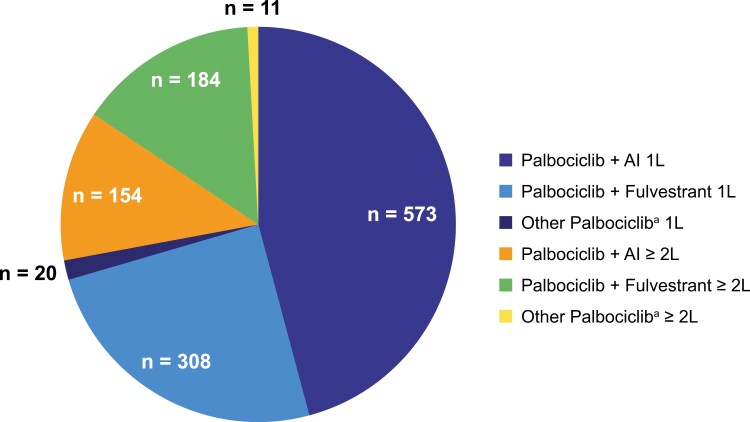
Treatment patterns at final data cutoff. ^a^Represents patients who, at study entry, received palbociclib with endocrine partners other than letrozole, anastrozole, exemestane, or fulvestrant or received palbociclib with no endocrine partners. Abbreviations: 1L, first-line; 2L, second-line; AI, aromatase inhibitor.

For the cohort included in this study, palbociclib-based therapy was initiated as 1L in 901 patients and as ≥2L in 349 patients (Figure 1). Regimens included palbociclib + AI (letrozole, anastrozole, or exemestane), palbociclib + fulvestrant, and other palbociclib (defined as palbociclib + another ET or palbociclib with no endocrine partner). Overall, 727 patients received palbociclib + AI, 492 patients received palbociclib + fulvestrant, and 31 patients received other palbociclib regimens. Owing to the infrequent use of other palbociclib regimens, our analyses and discussion herein focus on outcomes in patients receiving palbociclib + AI or palbociclib + fulvestrant, that together represent 97.5% of patients; data for patients receiving other palbociclib regimens are presented in Supplementary Tables S1-S3.

In the cohort that received palbociclib as 1L therapy (*n* = 901), 573 patients (63.6%) and 308 patients (34.2%) received AI and fulvestrant, respectively, as the endocrine partner; 10.3% (*n* = 70/680) had a DFI of ≤12 months, 5.4% (*n* = 37/680) had a DFI of >12 to ≤24 months, 9.9% (*n* = 67/680) had a DFI of >24 to ≤36 months, and 72.6% (*n* = 494/680) had a DFI of >36 months (Table 1).

In the cohort that received palbociclib as ≥2L therapy (*n* = 349), 154 patients (44.1%) and 184 patients (52.7%) received AI and fulvestrant, respectively, as the endocrine partner; 12.4% (*n* = 28/225) of patients had a DFI of ≤12 months, 4.4% (*n* = 10/225) had a DFI of >12 to ≤24 months, 9.3% (*n* = 21/225) had a DFI of >24 to ≤36 months, and 69.8% (*n* = 157/225) had a DFI of >36 months.

### Effectiveness outcomes

**Figure 2.**
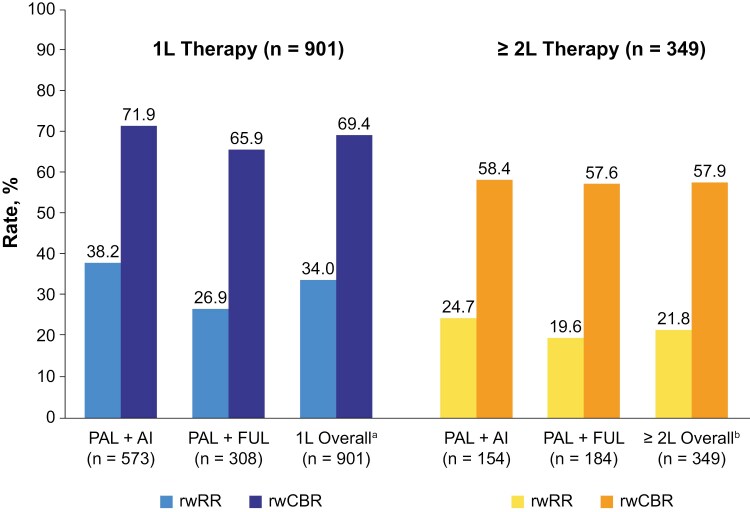
Real-world response rates and real-world clinical benefit rates by line of therapy and endocrine partner. ^a^Includes data from 20 patients who received 1L treatment with other palbociclib regimens (defined as palbociclib with no endocrine partners, or palbociclib with endocrine partners other than letrozole, anastrozole, fulvestrant, or exemestane), which are reported in Supplementary Table S2. ^b^Includes data from 11 patients who received ≥2L treatment with other palbociclib regimens (defined as palbociclib with no endocrine partners, or palbociclib with endocrine partners other than letrozole, anastrozole, fulvestrant, or exemestane), which are reported in Supplementary Table S2. Abbreviations: 1L, first-line; 2L, second-line; AI, aromatase inhibitor; FUL, fulvestrant; PAL, palbociclib; rwCBR, real-world clinical benefit rate; rwRR, real-world response rate.

rwRR and rwCBR by line of therapy and endocrine partner are summarized in Figure 2, and best responses are summarized in Supplementary Table S4. The overall rwRR and rwCBR with palbociclib + ET in the 1L setting was 34.0% and 69.4%, respectively. The rwRR (rwCBR) by ET partner in the 1L setting was 38.2% (71.9%) in the palbociclib + AI cohort and 26.9% (65.9%) in the palbociclib + fulvestrant cohort. In the ≥2L setting, the rwRR (rwCBR) was 21.8% (57.9%) in the overall cohort, 24.7% (58.4%) in the palbociclib + AI cohort, and 19.6% (57.6%) in the palbociclib + fulvestrant cohort. rwRR and rwCBR by line of therapy and endocrine partner are summarized for various patient subgroups in Table 2, including analyses by age, sex, race, menopausal status, number of disease sites, bone-only metastases, and visceral metastases. Results were generally consistent with the overall population, except for subgroups with low sample sizes (eg, male or premenopausal patients) that limit interpretation of the results.

**Table 2.** Real-world tumor response rates by subgroup.

Subgroup	1L therapy (*n* = 901^a^)						≥2L therapy (*n* = 349^a^)					
	PAL + AI (*n* = 573)			PAL + FUL (*n* = 308)			PAL + AI (*n* = 154)			PAL + FUL (*n* = 184)		
	*n*	rwRR, %	rwCBR, %	*n*	rwRR, %	rwCBR, %	*n*	rwRR, %	rwCBR, %	*n*	rwRR, %	rwCBR, %
Overall	573	38.2	71.9	308	26.9	65.9	154	24.7	58.4	184	19.6	57.6
Age												
<65 years	296	33.8	69.9	158	27.2	65.8	86	24.4	57.0	91	18.7	54.9
≥65 years	275	42.5	73.8	149	26.8	65.8	67	25.4	61.2	93	20.4	60.2
Sex												
Male	5	20.0	60.0	4	50.0	50.0	2	0	50.0	4	50.0	100.0
Female	568	38.4	72.0	304	26.6	66.1	152	25.0	58.6	180	18.9	56.7
BIPOC^b^												
Yes	115	36.5	75.7	79	25.3	70.9	40	10.0	70.0	38	18.4	63.2
No	438	38.6	71.5	215	27.9	65.1	107	27.1	52.3	142	19.7	56.3
Menopausal status												
Pre/perimenopausal	75	30.7	72.0	29	41.4	72.4	24	45.8	79.2	15	20.0	53.3
Postmenopausal	486	39.7	71.8	273	24.9	65.2	124	21.0	53.2	161	18.6	56.5
Number of disease sites												
1	284	37.3	73.6	140	22.9	65.0	65	27.7	69.2	66	13.6	50.0
≥2	264	39.4	71.2	150	30.7	67.3	81	22.2	48.1	107	23.4	60.7
Bone-only disease												
Yes	213	36.6	76.5	97	19.6	67.0	42	23.8	64.3	44	13.6	56.8
No	360	39.2	69.2	211	30.3	65.4	112	25.0	56.3	140	21.4	57.9
Visceral disease												
Yes	192	39.1	69.3	130	30.0	63.1	72	20.8	50.0	86	23.3	57.0
No	381	37.8	73.2	178	24.7	68.0	82	28.0	65.9	98	16.3	58.2

^a^Due to small sample sizes, subgroup analyses were not performed for patients who received treatment with other palbociclib regimens (defined as palbociclib with no endocrine partners, or palbociclib with endocrine partners other than letrozole, anastrozole, fulvestrant, or exemestane) in the 1L (*n* = 20) or ≥2L (*n* = 11) setting.

^b^BIPOC “no” defined as White and not Hispanic/Latino. BIPOC “yes” defined as all other race/ethnicity categories.

Abbreviations: 1L, first-line; 2L, second-line; AI, aromatase inhibitor; BIPOC, Black, Indigenous, and People of Color; FUL, fulvestrant; PAL, palbociclib; rwCBR, real-world clinical benefit rate; rwRR, real-world response rate.

For patients treated in the 1L setting, overall median rwPFS was 20.9 (95% CI, 18.7-24.7) months after a median follow-up duration of 35.4 (95% CI, 33.1-37.5) months. Median rwPFS was 25.8 (95% CI, 20.7-29.5) months in the 1L palbociclib + AI cohort and 17.1 (95% CI, 12.9-19.4) months in the 1L palbociclib + fulvestrant cohort (Table 3). For patients treated in the ≥2L setting, overall median rwPFS was 13.5 (95% CI, 10.6-17.1) months after a median follow-up duration of 35.8 (95% CI, 31.6-40.6) months. The median rwPFS was 13.2 (95% CI, 8.5-17.3) months in the ≥2L palbociclib + AI cohort and 13.5 (95% CI, 9.5-17.5) months in the ≥2L palbociclib + fulvestrant cohort. Table 4 shows rwPFS outcomes by line of therapy and endocrine partner for subgroups of interest. Consistently across lines of therapy and endocrine partners, rwPFS was numerically higher in patients with 1 disease site (vs those with ≥2 disease sites), without visceral disease (vs those with visceral disease), and with bone-only disease (vs those without bone-only disease). Otherwise, when small sample sizes were not a limiting factor, there were no notable differences in median rwPFS between the paired subgroups within each of the other baseline characteristic categories that remained consistent across different lines of therapy and endocrine partners.

**Table 3.** Real-world progression-free survival and overall survival by line of therapy and endocrine partner.

	1L therapy (*n* = 901)			≥2L therapy (*n* = 349)			Overall patients^a^^,^^b^ (*N* = 1250)
	PAL + AI (*n* = 573)	PAL + FUL (*n* = 308)	1L overall^a^ (*n* = 901)	PAL + AI (*n* = 154)	PAL + FUL (*n* = 184)	≥2L Overall^b^ (*n* = 349)	
rwPFS^c^							
Event, *n* (%)	286 (49.9)	178 (57.8)	473 (52.5)	89 (57.8)	128 (69.6)	224 (64.2)	697 (55.8)
Median (95% CI), months	25.8 (20.7-29.5)	17.1 (12.9-19.4)	20.9 (18.7-24.7)	13.2 (8.5-17.3)	13.5 (9.5-17.5)	13.5 (10.6-17.1)	18.8 (17.2-20.5)
**OS** ^d^							
Event, *n* (%)	199 (34.7)	122 (39.6)	329 (36.5)	68 (44.2)	103 (56.0)	176 (50.4)	505 (40.4)
Median (95% CI), months	53.3 (43.0-NE)	42.7 (34.6-55.0)	48.5 (42.0-NE)	42.2 (30.6-50.6)	32.1 (28.4-40.0)	37.2 (31.2-40.8)	42.3 (39.3-47.1)

^a^Includes data from 20 patients who received 1L treatment with other palbociclib regimens (defined as palbociclib with no endocrine partners, or palbociclib with endocrine partners other than letrozole, anastrozole, fulvestrant, or exemestane), which are reported in Supplementary Table S3.

^b^Includes data from 11 patients who received ≥2L treatment with other palbociclib regimens (defined as palbociclib with no endocrine partners, or palbociclib with endocrine partners other than letrozole, anastrozole, fulvestrant, or exemestane), which are reported in Supplementary Table S3.

^c^Median (95% CI) duration of follow-up for rwPFS analysis was 35.7 (33.2-37.5) months in the overall population, 35.4 (33.1-37.5) months in the 1L, and 35.8 (31.6-40.6) months in the ≥2L.

^d^Median (95% CI) duration of follow-up for OS analysis was 39.1 (37.9-40.3) months in the overall population, 38.6 (37.3-40.0) months in the 1L, and 41.0 (38.1-42.6) months in the ≥2L.

Abbreviations: 1L, first-line; 2L, second-line; AI, aromatase inhibitor; FUL, fulvestrant; NE, not estimable; OS, overall survival; PAL, palbociclib; rwPFS, real-world progression-free survival.

**Table 4.** Real-world progression-free survival by subgroup.

Subgroup	1L therapy (*n* = 901^a^)				≥2L therapy (*n* = 349^a^)			
	PAL + AI (*n* = 573)		PAL + FUL (*n* = 308)		PAL + AI (*n* = 154)		PAL + FUL (*n* = 184)	
	*n*	Median rwPFS (95% CI), mo.	*n*	Median rwPFS (95% CI), mo.	*n*	Median rwPFS (95% CI), mo.	*n*	Median rwPFS (95% CI), mo.
Overall	573	25.8 (20.7-29.5)	308	17.1 (12.9-19.4)	154	13.2 (8.5-17.3)	184	13.5 (9.5-17.5)
Age								
<65 years	296	21.7 (18.2-26.5)	158	15.9 (10.1-18.7)	86	13.1 (8.0-23.3)	91	13.5 (8.0-19.8)
≥65 years	275	29.5 (21.5-34.1)	149	18.3 (12.2-24.2)	67	13.2 (6.7-17.2)	93	13.7 (9.4-18.7)
Sex								
Male	5	30.1 (22.2-38.0)	4	19.4 (18.8-21.8)	2	NR (5.7-NE)	4	14.8 (7.4-42.8)
Female	568	25.7 (20.5-29.5)	304	16.8 (12.2-19.3)	152	13.2 (8.6-17.3)	180	13.5 (9.4-17.5)
BIPOC^b^								
Yes	115	20.3 (16.2-36.5)	79	24.2 (10.0-30.9)	40	13.2 (6.4-NE)	38	15.5 (8.1-37.9)
No	438	24.8 (20.4-29.0)	215	15.9 (12.1-18.7)	107	11.6 (7.4-17.1)	142	13.7 (9.0-17.5)
Menopausal status								
Pre/perimenopausal	75	15.4 (11.9-33.1)	29	28.6 (8.3-NE)	24	20.5 (10.0-NE)	15	12.0 (2.5-NE)
Postmenopausal	486	26.3 (21.3-30.4)	273	15.2 (11.8-18.6)	124	11.5 (6.7-16.8)	161	13.9 (9.5-17.5)
Number of disease sites								
1	284	29.0 (22.3-36.9)	140	18.2 (13.6-23.2)	65	17.3 (7.8-NE)	66	14.8 (4.7-24.0)
≥2	264	20.7 (17.9-26.7)	150	13.1 (8.7-19.4)	81	11.6 (6.1-16.8)	107	11.6 (8.7-17.2)
Bone-only disease								
Yes	213	28.1 (19.1-35.2)	97	17.4 (12.1-23.2)	42	14.3 (5.7-22.1)	44	19.8 (12.6-33.8)
No	360	25.2 (20.2-29.7)	211	16.5 (10.4-20.5)	112	13.2 (8.5-17.8)	140	11.6 (8.7-15.5)
Visceral disease								
Yes	192	23.2 (18.2-30.8)	130	15.9 (8.9-23.6)	72	11.6 (6.2-19.0)	86	10.8 (8.0-17.2)
No	381	27.2 (20.8-31.1)	178	17.4 (12.2-21.0)	82	14.3 (8.0-22.1)	98	16.1 (10.4-24.0)

^a^Due to small sample sizes, subgroup analyses were not performed for patients who received treatment with other palbociclib regimens (defined as palbociclib with no endocrine partners, or palbociclib with endocrine partners other than letrozole, anastrozole, fulvestrant, or exemestane) in the 1L (*n* = 20) or ≥2L (*n* = 11) setting.

^b^BIPOC “no” defined as White and not Hispanic/Latino. BIPOC “yes” defined as all other race/ethnicity categories.

Abbreviations: 1L, first-line; 2L, second-line; AI, aromatase inhibitor; BIPOC, Black, Indigenous, and People of Color; FUL, fulvestrant; mo, month; NE, not estimable; NR, not reached; PAL, palbociclib; rwPFS, real-world progression-free survival.

After a median follow-up duration of 38.6 (95% CI, 37.3-40.0) months, median OS was 48.5 (95% CI, 42.0-not estimable [NE]) months in the 1L setting overall, 53.3 (95% CI, 43.0-NE) months in the 1L palbociclib + AI cohort, and 42.7 (95% CI, 34.6-55.0) months in the 1L palbociclib + fulvestrant cohort (Table 3). After a median follow-up duration of 41.0 (95% CI, 38.1-42.6) months, median OS was 37.2 (95% CI, 31.2-40.8) months in the ≥2L setting overall, 42.2 (95% CI, 30.6-50.6) months in the ≥2L palbociclib + AI cohort, and 32.1 (95% CI, 28.4-40.0) months in the ≥2L palbociclib plus fulvestrant cohort. Table 5 shows OS outcomes by line of therapy and endocrine partner for subgroups of interest. Notably, median OS was not reached in several subgroups. When small sample size or unknown data were not limiting factors, there were no notable numerical differences in median OS between the paired subgroups within each baseline characteristic category that remained consistent across different lines of therapy and endocrine partners.

**Table 5.** Overall survival by subgroup.

	1L therapy (*n* = 901^a^)				≥2L therapy (*n* = 349^a^)			
	PAL + AI (*n* = 573)		PAL + FUL (*n* = 308)		PAL + AI (*n* = 154)		PAL + FUL (*n* = 184)	
Subgroup	*n*	Median OS (95% CI), mo.	*n*	Median OS (95% CI), mo.	*n*	Median OS (95% CI), mo.	*n*	Median OS (95% CI), mo.
Overall	573	53.3 (43.0-NE)	308	42.7 (34.6-55.0)	154	42.2 (30.6-50.6)	184	32.1 (28.4-40.0)
Age								
<65 years	296	48.8 (39.2-NE)	158	42.0 (34.6-55.0)	86	44.0 (30.6-NE)	91	30.1 (22.1-45.3)
≥65 years	275	NR (43.0-NE)	149	45.8 (28.8-NE)	67	37.9 (20.9-NE)	93	33.5 (28.5-40.1)
Sex								
Male	5	36.4 (35.6-NE)	4	28.8 (11.2-NE)	2	NR (7.1-NE)	4	NR (9.9-NE)
Female	568	53.3 (44.1-NE)	304	42.7 (34.6-55.0)	152	42.2 (30.6-50.6)	180	31.7 (27.0-39.5)
BIPOC^b^								
Yes	115	48.8 (36.6-NE)	79	49.1 (29.6-NE)	40	44.0 (24.2-NE)	38	39.0 (24.4-50.1)
No	438	53.3 (42.2-NE)	215	39.3 (34.0-NE)	107	40.6 (27.9-50.6)	142	31.7 (27.0-40.0)
Menopausal status								
Pre/perimenopausal	75	50.8 (30.8-NE)	29	49.1 (26.7-NE)	24	NR (40.1-NE)	15	26.5 (5.7-NE)
Postmenopausal	486	NR (44.1-NE)	273	42.0 (34.0-NE)	124	35.5 (22.0-48.2)	161	32.1 (28.5-40.0)
Number of disease sites								
1	284	NR (48.8-NE)	140	NR (35.5-NE)	65	44.0 (28.8-NE)	66	37.2 (25.8-NE)
≥2	264	42.2 (35.8-NE)	150	35.8 (29.6-45.8)	81	38.0 (21.0-48.2)	107	31.5 (24.4-37.9)
Bone-only disease								
Yes	213	50.8 (40.3-NE)	97	NR (34.6-NE)	42	44.0 (24.2-NE)	44	35.2 (27.0-NE)
No	360	53.3 (41.4-NE)	211	39.1 (32.7-47.1)	112	40.6 (29.4-50.6)	140	31.5 (25.2-39.5)
Visceral disease								
Yes	192	48.5 (36.5-NE)	130	35.8 (30.4-45.8)	72	40.1 (25.8-48.2)	86	30.6 (20.1-40.6)
No	381	NR (44.1-NE)	178	NR (34.6-NE)	82	44.0 (28.8-NE)	98	37.2 (28.4-42.6)

^a^Due to small sample sizes, subgroup analyses were not performed for patients who received treatment with other palbociclib regimens (defined as palbociclib with no endocrine partners, or palbociclib with endocrine partners other than letrozole, anastrozole, fulvestrant, or exemestane) in the 1L (*n* = 20) or ≥2L (*n* = 11) setting.

^b^BIPOC “no” defined as White and not Hispanic/Latino. BIPOC “yes” defined as all other race/ethnicity categories.

Abbreviations: 1L, first-line; 2L, second-line; AI, aromatase inhibitor; BIPOC, Black, Indigenous, and People of Color; FUL, fulvestrant; NE, not estimable; NR, not reached; OS, overall survival; PAL, palbociclib.

### Effectiveness outcomes: per-label analysis set

The effectiveness of palbociclib-based regimens was also assessed in the per-label population, representing 861 out of 1250 patients (68.9%) in the safety analysis set. Among these patients, 533 received 1L palbociclib + AI, 179 received 1L palbociclib + fulvestrant after prior ET for early breast cancer, and 149 received ≥ 2L palbociclib + fulvestrant after prior ET in any setting. Baseline demographic and disease characteristics for this population are presented in Supplementary Table S5.

rwRR (rwCBR) was 38.8% (73.2%) in the 1L palbociclib + AI cohort, 26.8% (69.3%) in the 1L palbociclib + fulvestrant cohort, and 18.8% (56.4%) in the ≥2L palbociclib + fulvestrant cohort (Supplementary Table S6). Median rwPFS was 25.5 (95% CI, 20.4-29.5) months, 18.7 (95% CI, 13.1-23.8) months, and 12.6 (95% CI, 9.0-18.7) months, and median OS was 50.8 (95% CI, 42.2-NE) months, 47.1 (95% CI, 35.8-NE) months, and 32.1 (95% CI, 28.5-40.1) months with per-label 1L palbociclib + AI, 1L palbociclib + fulvestrant, and ≥2L palbociclib + fulvestrant, respectively (Supplementary Table S7). Subgroup analyses of effectiveness outcomes in the per-label analysis set are presented in Supplementary Tables S8–10. Within each per-label treatment cohort, rwRR and rwCBR results were consistent with the overall population across subgroups, except for subgroups with low sample sizes (eg, male patients; Supplementary Table S8). For rwPFS and OS, when small sample size or unknown data were not limiting factors, there were no notable numerical differences in median survival between the paired subgroups within each baseline characteristic category that remained consistent across different lines of therapy and endocrine partners (Supplementary Tables S9 and S10).

## Discussion

In this prospective, multicenter, non-interventional study, we evaluated real-world treatment patterns and effectiveness outcomes in a heterogeneous group of patients with HR+/HER2− ABC receiving palbociclib in routine clinical practice. Overall, AIs were found to be the preferred endocrine partner to 1L palbociclib, with approximately two-thirds of patients receiving this combination in the 1L setting. This observation is consistent with the indication for palbociclib,^22^ but may also partly reflect patient preference for oral (AI) over injectable (fulvestrant) treatment.^31^ Similar proportions of patients received palbociclib + AI or palbociclib + fulvestrant as ≥2L therapy. Clinical outcome data for rwRR, rwCBR, rwPFS, and OS are also presented, and although POLARIS was not designed to compare effectiveness across therapy line or endocrine partner, as expected, numerically better outcomes were observed with palbociclib in the 1L setting relative to later lines. Subgroup analyses suggested effectiveness outcomes that were generally similar to the overall study population (with some exceptions, particularly where small numbers or missing data were a limitation [eg, male or premenopausal patients]) and served to confirm a generally consistent benefit of palbociclib + ET across a diverse range of patient subgroups.

Effectiveness outcomes observed in the overall population of our large, prospective, real-world study are generally consistent with those from clinical trials and other real-world studies that evaluated the use of palbociclib + ET in patients with HR+/HER2− ABC.^12,13,23-25,32,33^ Prior clinical studies evaluating the use of palbociclib + ET in patients with HR+/HER2−ABC included the PALOMA-2 and PALOMA-3 studies.^12,13,32,33^ In the PALOMA-2 study of 1L therapy for women with ER+/HER2− ABC, palbociclib + letrozole demonstrated a statistically significant improvement in median PFS (27.6 vs 14.5 months; hazard ratio [HR], 0.563; *P* < .0001) and a numerically higher median OS (53.9 vs 51.2 months; HR, 0.956; *P* = .3378), albeit not statistically significant, compared with placebo + letrozole.^13,32^ In the PALOMA-3 study of palbociclib + fulvestrant vs placebo + fulvestrant in women with HR+/HER2− MBC whose disease had progressed after prior ET, a statistically significant improvement in median PFS was achieved with palbociclib (9.5 vs 4.6 months; HR, 0.46; *P* < .0001), and a numerical, not statistically significant improvement in median OS was observed (34.9 vs 28.0 months; HR, 0.81; *P* = .09).^12,33^ A comparison of baseline demographics between POLARIS and the palbociclib arms of the PALOMA-2/3 trials showed comparable median ages (POLARIS, 64 years; PALOMA-2, 62 years; PALOMA-3, 57 years).^12,13^ While the majority of patients in all 3 studies were White, POLARIS enrolled a larger percentage of Black patients (11% vs 2% [PALOMA-2] and 6% [PALOMA-3]), whereas the PALOMA studies enrolled a larger percentage of Asian patients (15% [PALOMA-2] and 21% [PALOMA-3] vs 2%). In POLARIS, premenopausal (11.9%) and postmenopausal (86.6%) women as well as men (1.2%) were enrolled, while only postmenopausal women were enrolled in PALOMA-2 and postmenopausal (79%) and premenopausal (21%) women were enrolled in PALOMA-3. Differences in disease characteristics included more patients in POLARIS with bone-only disease (34% vs 23% [PALOMA-2] and 22% [PALOMA-3]), and fewer patients with visceral metastatic disease (42% vs 48% [PALOMA-2] and 59% [PALOMA-3]).

This analysis is similar to other recent real-world studies that retrospectively evaluated palbociclib + ET in patients with HR+/HER2− ABC using data from the Flatiron database,^23,24^ a single-institution database,^25^ or a pharmacy and medical records claims database.^26^ In the P-REALITY X study of the Flatiron database, 1L palbociclib + AI was associated with significantly prolonged median OS (49.1 vs 43.2 months; HR, 0.76; *P* < .0001) and rwPFS (19.3 vs 13.9 months; HR, 0.70; *P* < .0001) compared with AI after stabilized inverse probability treatment weighting (sIPTW).^23^ Another Flatiron retrospective analysis similarly demonstrated significantly prolonged median rwPFS (20.0 vs 11.9 months; HR, 0.58; *P* < .0001) and median OS (NE vs 43.1 months; HR, 0.66; *P* = .0002) with 1L palbociclib + letrozole vs letrozole alone after sIPTW.^24^ A real-world study at MD Anderson Cancer Center found significant improvement with 2L palbociclib + fulvestrant vs fulvestrant alone for both median rwPFS (10 vs 5 months, *P* < .0001) and median OS (32.3 vs 24.6 months; *P* = .0022) after propensity score matching (PSM).^25^ A significant benefit was observed with 1L palbociclib + AI vs AI alone in median rwPFS (17.4 vs 11.1 months; *P* = .0001) but not in median OS (44.3 vs 40.2 months; *P* = 1) after PSM; of note, the HR for OS in the 1L setting was 1 (95% CI, 0.81-1.23) after PSM and 0.79 (95% CI, 0.67-0.93) after IPTW. Lastly, pharmacy and medical claims data showed prolonged duration of treatment with palbociclib + ET vs ET alone in the 1L and 2L setting in men; notably, the US FDA expanded the palbociclib indication to include men based, in part, on these real-world data.^26^ Detailed analyses of treatment patterns, safety, quality of life, and clinical outcomes among male patients with HR+/HER2− ABC in the POLARIS study have recently been reported.^34^

Overall, a PFS benefit of adding palbociclib to ET in the 1L or ≥2L setting has been observed consistently across clinical and real-world studies^12,13,23-25,32,33^; however, a statistically significant OS benefit has only been observed in real-world studies to date.^23-25^ This discrepancy in OS findings could be partly attributed to the design of the PALOMA-2 and PALOMA-3 studies, which had greater statistical power to detect differences in PFS (the primary endpoint) than in OS (a secondary endpoint),^18,35^ along with variation in patient characteristics between real-world and clinical studies, as discussed previously.^23^ Certain real-world studies, such as P-REALITY X, had a much greater sample size than PALOMA-2, enabling greater statistical power to detect differences in OS. Moreover, enrollment in RCTs is confined to a cohort of patients that meets rigorous eligibility criteria, whereas real-world studies evaluate more heterogeneous patient populations, and consequently, may have results that are more generalizable to routine clinical practice. However, while observational real-world studies may provide greater statistical power, they can only help identify associations between an intervention and an outcome; RCTs provide a higher level of evidence for determining the efficacy (ie, causality) of an intervention when evaluating outcomes such as OS.

Strengths of the POLARIS study include the large size of the enrolled patient population that was analyzed (*N* = 1250), the diversity of participating academic and community institutions (>100 sites of care), the long follow-up duration, and the comprehensiveness of prospectively collected data, including evaluation of not only real-world effectiveness outcomes (reported herein) but also treatment sequencing, patient quality of life, longitudinal biomarkers, and geriatric-specific assessments,^30^ which will be reported in subsequent publications. Moreover, ongoing subgroup analyses are investigating effectiveness outcomes by other factors, including comorbidities,^36^ prior therapies, and dose modifications. However, interpretation of these real-world data is subject to a few limitations. Given the real-world setting of our study, tumor assessments did not occur on a fixed schedule as they would have within the confines of a clinical trial protocol; therefore, potential incomplete or missing data on disease assessments at follow-up visits must be acknowledged when interpreting results. Real-world tumor responses were determined by clinician interpretation based on radiology, laboratory evidence, clinical assessment, pathology, or clinical biomarkers and not solely on quantitative tumor measurements using RECIST criteria, as in clinical trials. Owing to the observational study design of POLARIS, patient selection and treatment assignment were determined by the treating physician in routine clinical practice. As a result, inherent selection bias and biases in prognostic factors, such as DFI, may have differed between the AI- and fulvestrant-treated patients and could have confounded the observed differences in outcomes. Finally, this study used convenience sampling, a type of non-probability sampling wherein investigators enroll patients according to their accessibility and availability^37^; thus, results may not be generalizable to the entire population with HR+/HER2− ABC.

## Conclusion

This prospective, multicenter, non-interventional study provides insight into treatment patterns and effectiveness outcomes for patients with HR+/HER2− ABC receiving palbociclib in real-world clinical practice. Effectiveness outcomes were generally consistent with those from RCTs and other real-world studies that evaluated the use of palbociclib + ET in patients with HR+/HER2− ABC. Overall, these real-world clinical outcome data in a heterogeneous population of patients with ABC add to the totality of clinical and real-world evidence that support the use of palbociclib + ET in patients with HR+/HER2− ABC.

## Supplementary material

Supplementary material is available at *The Oncologist* online.

oyae291_suppl_Supplementary_Figure_1_Tables_1-10

## Data Availability

Upon request, and subject to review, Pfizer will provide the data that support the findings of this study. Subject to certain criteria, conditions, and exceptions, Pfizer may also provide access to the related individual de-identified participant data. See https://www.pfizer.com/science/clinical-trials/data-and-results for more information.
