# Supplementary material for: Real-world effectiveness of palbociclib plus endocrine therapy in HR+/HER2− advanced breast cancer: final results from the POLARIS trial
Source: Oncologist. 2024 Oct 30;30(7):oyae291. doi: 10.1093/oncolo/oyae291 (PMC12311288; doi:10.1093/oncolo/oyae291)
Supplement: oyae291_suppl_Supplementary_Figure_1_Tables_1-10 [file oyae291_suppl_supplementary_figure_1_tables_1-10.docx]

**Supplementary Table 1. Baseline demographic and disease characteristics for patients treated with other palbociclib regimens^a^**

| **Characteristic** | **1L**  **(n = 20)** | **≥ 2L**  **(n = 11)** |
| --- | --- | --- |
| Age |  |  |
| n (missing) | 20 (0) | 11 (0) |
| Median (range), years | 62.5 (38–77) | 61.0 (34–78) |
| Distribution, n (%) |  |  |
| < 50 years | 6 (30.0) | 2 (18.2) |
| ≥ 50–< 70 years | 8 (40.0) | 8 (72.7) |
| ≥ 70 years | 6 (30.0) | 1 (9.1) |
| Sex, n (%) |  |  |
| Male | 0 | 0 |
| Female | 20 (100.0) | 11 (100.0) |
| Race, n (%) |  |  |
| White | 14 (70.0) | 9 (81.8) |
| Black/African American | 4 (20.0) | 1 (9.1) |
| Asian | 0 | 0 |
| American Indian/Alaska Native | 0 | 1 (9.1) |
| Native Hawaiian/other Pacific Islander | 0 | 0 |
| Other | 0 | 0 |
| Not reported or missing | 2 (10.0) | 0 |
| Ethnicity, n (%) |  |  |
| Not Hispanic/Latino | 14 (70.0) | 9 (81.8) |
| Hispanic/Latino | 5 (25.0) | 1 (9.1) |
| Not reported or missing | 1 (5.0) | 1 (9.1) |
| Menopause status |  |  |
| n (not applicable) | 20 (0) | 11 (0) |
| Distribution, n (%) |  |  |
| Post-menopausal | 17 (85.0) | 9 (81.8) |
| Pre- or peri-menopausal | 3 (15.0) | 1 (9.1) |
| Unknown | 0 | 1 (9.1) |
| Stage of diagnosis at study enrollment, n (%) |  |  |
| Locally advanced (stage III) | 1 (5.0) | 2 (18.2) |
| Metastatic (stage IV) | 19 (95.0) | 9 (81.8) |
| Not reported | 0 | 0 |
| Molecular subtype at most recent recurrence diagnosis prior to enrollment (or initial diagnosis, if no recurrence), n (%) |  |  |
| HR+/HER2– | 20 (100.0) | 9 (81.8) |
| HR–/HER2– | 0 | 0 |
| HR+/HER2+ | 0 | 1 (9.1) |
| HR–/HER2+ | 0 | 0 |
| Unknown | 0 | 1 (9.1) |
| Not reported | 0 | 0 |
| Disposition of diagnosis at study enrollment, n (%) |  |  |
| Recurrent from earlier stage, stages 0−III | 14 (70.0) | 6 (54.5) |
| *De novo*, newly diagnosed stage IV | 6 (30.0) | 4 (36.4) |
| Not reported | 0 | 1 (9.1) |
| Time from ABC/MBC diagnosis to enrollment |  |  |
| n (missing) | 20 (0) | 11 (0) |
| Median (range), months | 0.8 (0–24) | 38.3 (3–175) |
| Distribution, n (%) |  |  |
| ≤ 1 month | 12 (60.0) | 0 |
| > 1−≤ 2 months | 6 (30.0) | 0 |
| > 2−≤ 6 months | 1 (5.0) | 2 (18.2) |
| > 6 months | 1 (5.0) | 9 (81.8) |
| Disease-free interval^b^ |  |  |
| n (missing) | 14 (0) | 7 (0) |
| Median (range), months | 52.9 (1−145) | 125.3 (0−177) |
| Distribution, n (%) |  |  |
| No disease-free interval^c^ | 0 | 1 (14.3) |
| ≤ 12 months | 2 (14.3) | 1 (14.3) |
| > 12−≤ 24 months | 2 (14.3) | 0 |
| > 24−≤ 36 months | 2 (14.3) | 0 |
| > 36 months | 8 (57.1) | 5 (71.4) |
| Sites of distant metastases at MBC diagnosis^d^ |  |  |
| n (missing) | 19 (0) | 9 (0) |
| Median (range) number of sites | 2.0 (1−4) | 2.0 (1−4) |
| Bone involvement at MBC diagnosis^d^, n (%) |  |  |
| Bone + other metastases | 6 (31.6) | 5 (55.6) |
| Bone-only | 9 (47.4) | 0 |
| Visceral disease^e^ at MBC diagnosis^d^, n (%) |  |  |
| Yes | 6 (31.6) | 8 (88.9) |
| No | 13 (68.4) | 1 (11.1) |

^a^Other palbociclib regimens defined as palbociclib with no endocrine partners, or palbociclib with endocrine partners other than letrozole, anastrozole, fulvestrant, or exemestane.

^b^Disease-free interval from first diagnosis of breast cancer to first onset of relapse/recurrent disease among patients with non-missing dates. Patients with initial breast cancer diagnosis of MBC (i.e., *de novo* metastatic disease) are excluded from the disease-free interval calculation.

^c^No disease-free interval indicates that the initial diagnosis date is the same as ABC/MBC diagnosis date in patients with non-MBC initial diagnosis.

^d^Among patients with metastatic disease at study enrollment.

^e^Metastases of the brain, liver, and/or lung/pleura.

1L, first-line; 2L, second-line; ABC, advanced breast cancer; MBC, metastatic breast cancer; N/A, not applicable.

**Supplementary Table 2. Real-world tumor response rates for patients treated with other palbociclib regimens^a^**

| **Response** | **1L**  **(n = 20)** | **≥ 2L**  **(n = 11)** |
| --- | --- | --- |
| Best response, n (%) |  |  |
| rwCR | 0 | 1 (9.1) |
| rwPR | 4 (20.0) | 1 (9.1) |
| rwSD | 4 (20.0) | 6 (54.5) |
| rwPD | 5 (25.0) | 2 (18.2) |
| Indeterminate | 7 (35.0) | 1 (9.1) |
| rwRR,^b^ n (%) | 4 (20.0) | 2 (18.2) |
| rwCBR,^c^ n (%) | 10 (50.0) | 6 (54.5) |

^a^Other palbociclib regimens defined as palbociclib with no endocrine partners, or palbociclib with endocrine partners other than letrozole, anastrozole, fulvestrant, or exemestane.

^b^rwRR defined as the proportion of patients with a best response of either rwCR or rwPR in patients with at least one disease assessment during the respective line of therapy (after palbociclib initiation and before the start date of the next line of treatment).

^c^rwCBR defined as the proportion of patients with a best response of rwCR or rwPR at any time, or rwSD for at least 24 weeks, in patients with at least one disease assessment during the respective line of therapy (after palbociclib initiation and before the start date of the next line of treatment).

1L, first-line; 2L, second-line; rwCBR, real-world clinical benefit rate; rwCR, real-world complete response; rwPD, real-world progressive disease; rwPR, real-world partial response; rwRR, real-world response rate; rwSD, real-world stable disease.

**Supplementary Table 3. Real-world progression-free survival and overall survival for patients treated with other palbociclib regimens^a^**

|  | **1L**  **(n = 20)** | **≥ 2L**  **(n = 11)** |
| --- | --- | --- |
| **rwPFS** |  |  |
| Event, n (%) | 9 (45.0) | 7 (63.6) |
| Median (95% CI), months | 10.9 (2.4–NE) | 20.7 (3.8−NE) |
| **OS** |  |  |
| Event, n (%) | 8 (40.0) | 5 (45.5) |
| Median (95% CI), months | 35.6 (11.7−NE) | 40.8 (12.5−NE) |

^a^Other palbociclib regimens defined as palbociclib with no endocrine partners, or palbociclib with endocrine partners other than letrozole, anastrozole, fulvestrant, or exemestane.

1L, first-line; 2L, second-line; CI, confidence interval; NE, not estimable; OS, overall survival; rwPFS, real-world progression-free survival.

**Supplementary Table 4. Real-world tumor response rates by line of therapy and endocrine partner**

|  | **1L Therapy (n = 901)** | | | **≥ 2L Therapy (n = 349)** | | | **Overall Patients^a,b^**  **(N = 1250)** |
| --- | --- | --- | --- | --- | --- | --- | --- |
|  | **PAL + AI**  **(n = 573)** | **PAL + FUL**  **(n = 308)** | **1L Overall^a^**  **(n = 901)** | **PAL + AI**  **(n = 154)** | **PAL + FUL**  **(n = 184)** | **≥ 2L Overall^b^**  **(n = 349)** |  |
| Best response, n (%) |  |  |  |  |  |  |  |
| rwCR | 45 (7.9) | 14 (4.5) | 59 (6.5) | 5 (3.2) | 7 (3.8) | 13 (3.7) | 72 (5.8) |
| rwPR | 174 (30.4) | 69 (22.4) | 247 (27.4) | 33 (21.4) | 29 (15.8) | 63 (18.1) | 310 (24.8) |
| rwSD | 218 (38.0) | 127 (41.2) | 349 (38.7) | 73 (47.4) | 71 (38.6) | 150 (43.0) | 499 (39.9) |
| rwPD | 74 (12.9) | 61 (19.8) | 140 (15.5) | 29 (18.8) | 52 (28.3) | 83 (23.8) | 223 (17.8) |
| Indeterminate | 62 (10.8) | 37 (12.0) | 106 (11.8) | 14 (9.1) | 25 (13.6) | 40 (11.5) | 146 (11.7) |
| rwRR,^c^ n (%) | 219 (38.2) | 83 (26.9) | 306 (34.0) | 38 (24.7) | 36 (19.6) | 76 (21.8) | 382 (30.6) |
| rwCBR,^d^ n (%) | 412 (71.9) | 203 (65.9) | 625 (69.4) | 90 (58.4) | 106 (57.6) | 202 (57.9) | 827 (66.2) |

^a^Includes data from 20 patients who received 1L treatment with other palbociclib regimens (defined as palbociclib with no endocrine partners, or palbociclib with endocrine partners other than letrozole, anastrozole, fulvestrant, or exemestane), which are reported in Supplementary Table 2.

^b^Includes data from 11 patients who received ≥ 2L treatment with other palbociclib regimens (defined as palbociclib with no endocrine partners, or palbociclib with endocrine partners other than letrozole, anastrozole, fulvestrant, or exemestane), which are reported in Supplementary Table 2.

^c^rwRR defined as the proportion of patients with a best response of either rwCR or rwPR in patients with at least one disease assessment during the respective line of therapy (after palbociclib initiation and before the start date of the next line of treatment).

^d^rwCBR defined as the proportion of patients with a best response of rwCR or rwPR at any time, or rwSD for at least 24 weeks, in patients with at least one disease assessment during the respective line of therapy (after palbociclib initiation and before the start date of the next line of treatment).

1L, first-line; 2L, second-line; AI, aromatase inhibitor; FUL, fulvestrant; PAL, palbociclib; rwCBR, real-world clinical benefit rate; rwCR, real-world complete response; rwPD, real-world progressive disease; rwPR, real-world partial response; rwRR, real-world response rate; rwSD, real-world stable disease.

**Supplementary Table 5. Patient baseline demographic and disease characteristics (per-label analysis set)**

|  | **Per Label**  **1L PAL + AI**  **(n = 533)** | **Per Label**  **1L PAL + FUL**  **(n = 179)** | **Per Label**  **≥ 2L PAL + FUL**  **(n = 149)** |
| --- | --- | --- | --- |
| Age |  |  |  |
| n (missing) | 533 (0) | 178 (1) | 149 (0) |
| Median (range), years | 64.0 (22–97) | 66.0 (29–91) | 65.0 (36–92) |
| Distribution, n (%) |  |  |  |
| < 50 years | 79 (14.8) | 19 (10.7) | 14 (9.4) |
| ≥ 50–< 70 years | 293 (55.0) | 84 (47.2) | 78 (52.3) |
| ≥ 70 years | 161 (30.2) | 75 (42.1) | 57 (38.3) |
| Sex, n (%) |  |  |  |
| Male | 5 (0.9) | 3 (1.7) | 4 (2.7) |
| Female | 528 (99.1) | 176 (98.3) | 145 (97.3) |
| Race, n (%) |  |  |  |
| White | 437 (82.0) | 150 (83.8) | 122 (81.9) |
| Black/African American | 66 (12.4) | 17 (9.5) | 16 (10.7) |
| Asian | 6 (1.1) | 2 (1.1) | 3 (2.0) |
| American Indian/Alaska Native | 3 (0.6) | 2 (1.1) | 1 (0.7) |
| Native Hawaiian/other Pacific Islander | 2 (0.4) | 1 (0.6) | 0 |
| Other | 8 (1.5) | 3 (1.7) | 4 (2.7) |
| Not reported or missing | 11 (2.1) | 4 (2.2) | 3 (2.0) |
| Ethnicity, n (%) |  |  |  |
| Not Hispanic/Latino | 487 (91.4) | 151 (84.4) | 137 (91.9) |
| Hispanic/Latino | 29 (5.4) | 23 (12.8) | 9 (6.0) |
| Not reported or missing | 17 (3.2) | 5 (2.8) | 3 (2.0) |
| Menopause status |  |  |  |
| n (not applicable) | 528 (5) | 176 (3) | 145 (4) |
| Distribution, n (%) |  |  |  |
| Post-menopausal | 450 (85.2) | 163 (92.6) | 131 (90.3) |
| Pre- or peri-menopausal | 73 (13.8) | 12 (6.8) | 10 (6.9) |
| Unknown | 5 (0.9) | 1 (0.6) | 4 (2.8) |
| Stage of diagnosis at study enrollment, n (%) |  |  |  |
| Locally advanced (stage III) | 21 (3.9) | 8 (4.5) | 8 (5.4) |
| Metastatic (stage IV) | 511 (95.9) | 170 (95.0) | 141 (94.6) |
| Not reported | 1 (0.2) | 1 (0.6) | 0 |
| Molecular subtype at most recent recurrence diagnosis prior to enrollment (or initial diagnosis, if no recurrence), n (%) |  |  |  |
| HR+/HER2– | 533 (100) | 179 (100) | 149 (100) |
| Disposition of diagnosis at study enrollment, n (%) |  |  |  |
| Recurrent from earlier stage, stages 0−III | 326 (61.2) | 175 (97.8) | 88 (59.1) |
| *De novo*, newly diagnosed stage IV | 185 (34.7) | 2 (1.1)^a^ | 50 (33.6) |
| Not reported | 22 (4.1) | 2 (1.1) | 11 (7.4) |
| Time from ABC/MBC diagnosis to enrollment |  |  |  |
| n (missing) | 530 (3) | 178 (1) | 149 (0) |
| Median (range), months | 0.9 (0–193) | 0.9 (0–52) | 32.5 (1–191) |
| Distribution, n (%) |  |  |  |
| ≤ 1 month | 299 (56.4) | 106 (59.6) | 1 (0.7) |
| > 1−≤ 2 months | 143 (27.0) | 46 (25.8) | 6 (4.0) |
| > 2−≤ 6 months | 60 (11.3) | 12 (6.7) | 7 (4.7) |
| > 6 months | 28 (5.3) | 14 (7.9) | 135 (90.6) |
| Disease-free interval^b^ |  |  |  |
| n (missing) | 345 (3) | 176 (1) | 99 (0) |
| Median (range), months | 82.9 (0–435) | 68.0 (0–371) | 86.7 (0–410) |
| Distribution, n (%) |  |  |  |
| No disease-free interval^c^ | 8 (2.3) | 1 (0.6) | 5 (5.1) |
| ≤ 12 months | 53 (15.4) | 4 (2.3) | 10 (10.1) |
| > 12−≤ 24 months | 19 (5.5) | 11 (6.3) | 3 (3.0) |
| > 24−≤ 36 months | 23 (6.7) | 26 (14.8) | 7 (7.1) |
| > 36 months | 242 (70.1) | 134 (76.1) | 74 (74.7) |
| Sites of distant metastases at MBC diagnosis^d^ |  |  |  |
| n (missing) | 511 (0) | 170 (0) | 141 (0) |
| Median (range) number of sites | 1.0 (1–10) | 1.0 (1–10) | 2.0 (1–7) |
| Bone involvement at MBC diagnosis^d^, n (%) |  |  |  |
| Bone + other metastases | 193 (37.8) | 61 (35.9) | 77 (54.6) |
| Bone-only | 199 (38.9) | 62 (36.5) | 38 (27.0) |
| Visceral disease^e^ at MBC diagnosis^d^, n (%) |  |  |  |
| Yes | 181 (35.4) | 80 (47.1) | 70 (49.6) |
| No | 330 (64.6) | 90 (52.9) | 71 (50.4) |

^a^Patients were reported on the case report form as having “de novo” metastatic disease, but records of anticancer systemic treatments given prior to the date of metastatic diagnosis were indicative of an earlier diagnosis.

^b^Disease-free interval from first diagnosis of breast cancer to first onset of relapse/recurrent disease among patients with non-missing dates. Patients with initial breast cancer diagnosis of MBC (i.e., *de novo* metastatic disease) are excluded from the disease-free interval calculation.

^c^No disease-free interval indicates that the initial diagnosis date is the same as ABC/MBC diagnosis date in patients with non-MBC initial diagnosis.

^d^Among patients with metastatic disease at study enrollment.

^e^Metastases of the brain, liver, and/or lung/pleura.

1L, first-line; 2L, second-line; ABC, advanced breast cancer; AI, aromatase inhibitor; FUL, fulvestrant; MBC, metastatic breast cancer; N/A, not applicable; PAL, palbociclib.

**Supplementary Table 6. Real-world tumor response rates by line of therapy and endocrine partner (per-label analysis set)**

|  | **Per Label**  **1L PAL + AI**  **(n = 533)** | **Per Label**  **1L PAL + FUL**  **(n = 179)** | **Per Label**  **≥ 2L PAL + FUL**  **(n = 149)** |
| --- | --- | --- | --- |
| Best response, n (%) |  |  |  |
| rwCR | 42 (7.9) | 6 (3.4) | 4 (2.7) |
| rwPR | 165 (31.0) | 42 (23.5) | 24 (16.1) |
| rwSD | 201 (37.7) | 82 (45.8) | 56 (37.6) |
| rwPD | 66 (12.4) | 31 (17.3) | 43 (28.9) |
| Indeterminate | 59 (11.1) | 18 (10.1) | 22 (14.8) |
| rwRR,^a^ n (%) | 207 (38.8) | 48 (26.8) | 28 (18.8) |
| rwCBR,^b^ n (%) | 390 (73.2) | 124 (69.3) | 84 (56.4) |

^a^rwRR defined as the proportion of patients with a best response of either rwCR or rwPR in patients with at least one disease assessment during the respective line of therapy (after palbociclib initiation and before the start date of the next line of treatment).

^b^rwCBR defined as the proportion of patients with a best response of rwCR or rwPR at any time, or rwSD for at least 24 weeks, in patients with at least one disease assessment during the respective line of therapy (after palbociclib initiation and before the start date of the next line of treatment).

1L, first-line; 2L, second-line; AI, aromatase inhibitor; FUL, fulvestrant; PAL, palbociclib; rwCBR, real-world clinical benefit rate; rwCR, real-world complete response; rwPD, real-world progressive disease; rwPR, real-world partial response; rwRR, real-world response rate; rwSD, real-world stable disease.

**Supplementary Table 7. Real-world progression-free survival and overall survival by line of therapy and endocrine partner (per-label analysis set)**

|  | **Per Label**  **1L PAL + AI**  **(n = 533)** | **Per Label**  **1L PAL + FUL**  **(n = 179)** | **Per Label**  **≥ 2L PAL + FUL**  **(n = 149)** |
| --- | --- | --- | --- |
| **rwPFS** |  |  |  |
| Event, n (%) | 267 (50.1) | 100 (55.9) | 105 (70.5) |
| Median (95% CI), months | 25.5 (20.4–29.5) | 18.7 (13.1–23.8) | 12.6 (9.0–18.7) |
| **OS** |  |  |  |
| Event, n (%) | 191 (35.8) | 64 (35.8) | 81 (54.4) |
| Median (95% CI), months | 50.8 (42.2–NE) | 47.1 (35.8–NE) | 32.1 (28.5–40.1) |

1L, first-line; 2L, second-line; AI, aromatase inhibitor; CI, confidence interval; FUL, fulvestrant; NE, not estimable; OS, overall survival; PAL, palbociclib; rwPFS, real-world progression-free survival.

**Supplementary Table 8. Real-world tumor response rates by subgroup (per-label analysis set)**

|  | **Per Label**  **1L PAL + AI**  **(n = 533)** | | | **Per Label**  **1L PAL + FUL**  **(n = 179)** | | | **Per Label**  **≥ 2L PAL + FUL**  **(n = 149)** | | | |
| --- | --- | --- | --- | --- | --- | --- | --- | --- | --- | --- |
|  | **n** | **rwRR,**  **%** | **rwCBR,**  **%** | **n** | **rwRR,**  **%** | **rwCBR,**  **%** | **n** | | **rwRR,**  **%** | **rwCBR,**  **%** |
| Overall | 533 | 38.8 | 73.2 | 179 | 26.8 | 69.3 | 149 | 18.8 | | 56.4 |
| Age |  |  |  |  |  |  |  |  | |  |
| < 65 years | 284 | 33.8 | 70.8 | 86 | 26.7 | 70.9 | 70 | 20.0 | | 54.3 |
| ≥ 65 years | 249 | 44.6 | 75.9 | 92 | 27.2 | 67.4 | 79 | 17.7 | | 58.2 |
| Sex |  |  |  |  |  |  |  |  | |  |
| Male | 5 | 20.0 | 60.0 | 3 | 66.7 | 66.7 | 4 | 50.0 | | 100.0 |
| Female | 528 | 39.0 | 73.3 | 176 | 26.1 | 69.3 | 145 | 17.9 | | 55.2 |
| BIPOC^a^ |  |  |  |  |  |  |  |  | |  |
| Yes | 107 | 33.6 | 73.8 | 46 | 21.7 | 76.1 | 30 | 16.7 | | 63.3 |
| No | 408 | 40.0 | 73.3 | 127 | 29.1 | 66.9 | 115 | 19.1 | | 54.8 |
| Menopausal status |  |  |  |  |  |  |  |  | |  |
| Pre/perimenopausal | 73 | 30.1 | 72.6 | 12 | 33.3 | 66.7 | 10 | 30.0 | | 50.0 |
| Postmenopausal | 450 | 40.4 | 73.3 | 163 | 25.8 | 69.3 | 131 | 16.8 | | 55.0 |
| Number of disease sites |  |  |  |  |  |  |  |  | |  |
| 1 | 263 | 38.4 | 74.9 | 87 | 19.5 | 69.0 | 51 | 11.8 | | 45.1 |
| ≥ 2 | 248 | 39.9 | 72.6 | 83 | 33.7 | 69.9 | 90 | 23.3 | | 61.1 |
| Bone-only disease |  |  |  |  |  |  |  |  | |  |
| Yes | 199 | 37.2 | 77.9 | 62 | 14.5 | 67.7 | 38 | 10.5 | | 50.0 |
| No | 334 | 39.8 | 70.4 | 117 | 33.3 | 70.1 | 111 | 21.6 | | 58.6 |
| Visceral disease |  |  |  |  |  |  |  |  | |  |
| Yes | 181 | 38.1 | 68.5 | 80 | 28.8 | 66.3 | 70 | 22.9 | | 57.1 |
| No | 352 | 39.2 | 75.6 | 99 | 25.3 | 71.7 | 79 | 15.2 | | 55.7 |

^a^BIPOC “no” defined as White and not Hispanic/Latino. BIPOC “yes” defined as all other race/ethnicity categories.

1L, first-line; 2L, second-line; AI, aromatase inhibitor; BIPOC, Black, Indigenous, and People of Color; FUL, fulvestrant; NE, not estimable; PAL, palbociclib; rwCBR, real-world clinical benefit rate; rwRR, real-world response rate.

**Supplementary Table 9. Real-world progression-free survival by subgroup (per-label analysis set)**

|  | **Per Label**  **1L PAL + AI**  **(n = 533)** | | **Per Label**  **1L PAL + FUL**  **(n = 179)** | | **Per Label**  **≥ 2L PAL + FUL**  **(n = 149)** | |
| --- | --- | --- | --- | --- | --- | --- |
|  | **n** | **Median rwPFS**  **(95% CI), mo.** | **n** | **Median rwPFS**  **(95% CI), mo.** | **n** | **Median rwPFS**  **(95% CI), mo.** |
| Overall | 533 | 25.5 (20.4–29.5) | 179 | 18.7 (13.1–23.8) | 149 | 12.6 (9.0–18.7) |
| Age |  |  |  |  |  |  |
| < 65 years | 284 | 20.9 (18.2–26.5) | 86 | 17.1 (9.4–23.6) | 70 | 13.5 (8.3–29.3) |
| ≥ 65 years | 249 | 30.4 (21.5–36.9) | 92 | 21.0 (12.2–30.2) | 79 | 12.0 (7.4–18.6) |
| Sex |  |  |  |  |  |  |
| Male | 5 | 30.1 (22.2–38.0) | 3 | 20.6 (19.4–21.8) | 4 | 14.8 (7.4–42.8) |
| Female | 528 | 25.2 (20.3–29.4) | 176 | 18.6 (13.1–24.2) | 145 | 12.6 (9.0–18.6) |
| BIPOC^a^ |  |  |  |  |  |  |
| Yes | 107 | 20.2 (16.1–33.9) | 46 | 23.6 (10.2–40.3) | 30 | 16.5 (8.1–37.9) |
| No | 408 | 24.8 (20.2–29.0) | 127 | 17.4 (12.2–23.8) | 115 | 12.0 (9.0–18.7) |
| Menopausal status |  |  |  |  |  |  |
| Pre/perimenopausal | 73 | 16.4 (11.9–33.1) | 12 | NR (2.9–NE) | 10 | 16.9 (2.1–NE) |
| Postmenopausal | 450 | 26.2 (20.9–30.4) | 163 | 18.6 (13.0–23.8) | 131 | 13.2 (9.0–18.7) |
| Number of disease sites |  |  |  |  |  |  |
| 1 | 263 | 29.0 (22.1–35.2) | 87 | 19.5 (15.9–27.2) | 51 | 11.6 (4.7–24.0) |
| ≥ 2 | 248 | 21.0 (18.2–27.2) | 83 | 13.1 (8.8–23.8) | 90 | 11.6 (8.3–17.5) |
| Bone-only disease |  |  |  |  |  |  |
| Yes | 199 | 28.1 (18.5–35.2) | 62 | 18.7 (15.2–27.2) | 38 | 19.5 (5.8–25.8) |
| No | 334 | 25.2 (19.6–29.7) | 117 | 18.3 (9.1–24.9) | 111 | 11.6 (8.3–16.1) |
| Visceral disease |  |  |  |  |  |  |
| Yes | 181 | 21.0 (16.8–26.5) | 80 | 19.3 (9.0–26.1) | 70 | 10.8 (6.7–19.8) |
| No | 352 | 27.2 (20.8–31.1) | 99 | 18.6 (12.2–27.2) | 79 | 15.2 (8.7–21.9) |

^a^BIPOC “no” defined as White and not Hispanic/Latino. BIPOC “yes” defined as all other race/ethnicity categories.

1L, first-line; 2L, second-line; AI, aromatase inhibitor; BIPOC, Black, Indigenous, and People of Color; CI, confidence interval; FUL, fulvestrant; mo, month; NE, not estimable; NR, not reached; PAL, palbociclib; rwPFS, real world progression-free survival.

**Supplementary Table 10. Overall survival by subgroup (per-label analysis set)**

|  | **Per Label**  **1L PAL + AI**  **(n = 533)** | | **Per Label**  **1L PAL + FUL**  **(n = 179)** | | **Per Label**  **≥ 2L PAL + FUL**  **(n = 149)** | |
| --- | --- | --- | --- | --- | --- | --- |
| **Subgroup** | **n** | **Median OS**  **(95% CI), mo.** | **n** | **Median OS**  **(95% CI), mo.** | **n** | **Median OS**  **(95% CI), mo.** |
| Overall | 533 | 50.8 (42.2–NE) | 179 | 47.1 (35.8–NE) | 149 | 32.1 (28.5–40.1) |
| Age |  |  |  |  |  |  |
| < 65 years | 284 | 45.6 (38.4–NE) | 86 | 47.1 (34.6–NE) | 70 | 29.4 (23.1–48.0) |
| ≥ 65 years | 249 | NR (42.4–NE) | 92 | NR (30.4–NE) | 79 | 33.5 (28.5–40.1) |
| Sex |  |  |  |  |  |  |
| Male | 5 | 36.4 (35.6–NE) | 3 | NR (11.2–NE) | 4 | NR (9.9–NE) |
| Female | 528 | 50.8 (42.2–NE) | 176 | 47.1 (35.8–NE) | 145 | 31.7 (28.4–40.0) |
| BIPOC^a^ |  |  |  |  |  |  |
| Yes | 107 | 48.8 (35.5–NE) | 46 | 55.0 (29.6–NE) | 30 | 41.6 (13.5–NE) |
| No | 408 | 50.8 (40.5–NE) | 127 | 46.9 (35.5–NE) | 115 | 31.7 (28.4–40.0) |
| Menopausal status |  |  |  |  |  |  |
| Pre/perimenopausal | 73 | 50.8 (30.8–NE) | 12 | NR (8.4–NE) | 10 | 27.0 (3.5–NE) |
| Postmenopausal | 450 | 53.3 (42.4–NE) | 163 | 47.1 (35.8–NE) | 131 | 31.7 (28.5–40.1) |
| Number of disease sites |  |  |  |  |  |  |
| 1 | 263 | NR (48.8–NE) | 87 | NR (NE–NE) | 51 | 37.2 (25.8–NE) |
| ≥ 2 | 248 | 41.4 (35.7–NE) | 83 | 39.1 (30.4–55.0) | 90 | 31.5 (23.1–39.5) |
| Bone-only disease |  |  |  |  |  |  |
| Yes | 199 | 50.8 (39.3–NE) | 62 | NR (35.5–NE) | 38 | 33.5 (25.8–NE) |
| No | 334 | 53.3 (38.7–NE) | 117 | 45.8 (34.0–NE) | 111 | 31.7 (26.3–41.6) |
| Visceral disease |  |  |  |  |  |  |
| Yes | 181 | 42.2 (35.8–NE) | 80 | 39.1 (29.6–55.0) | 70 | 31.5 (19.2–42.8) |
| No | 352 | NR (43.0–NE) | 99 | NR (NE–NE) | 79 | 37.2 (28.4–42.6) |

^a^BIPOC “no” defined as White and not Hispanic/Latino. BIPOC “yes” defined as all other race/ethnicity categories.

1L, first-line; 2L, second-line; AI, aromatase inhibitor; BIPOC, Black, Indigenous, and People of Color; CI, confidence interval; FUL, fulvestrant; NE, not estimable; NR, not reached; OS, overall survival; PAL, palbociclib.

**Supplementary Figure 1. POLARIS patient disposition**


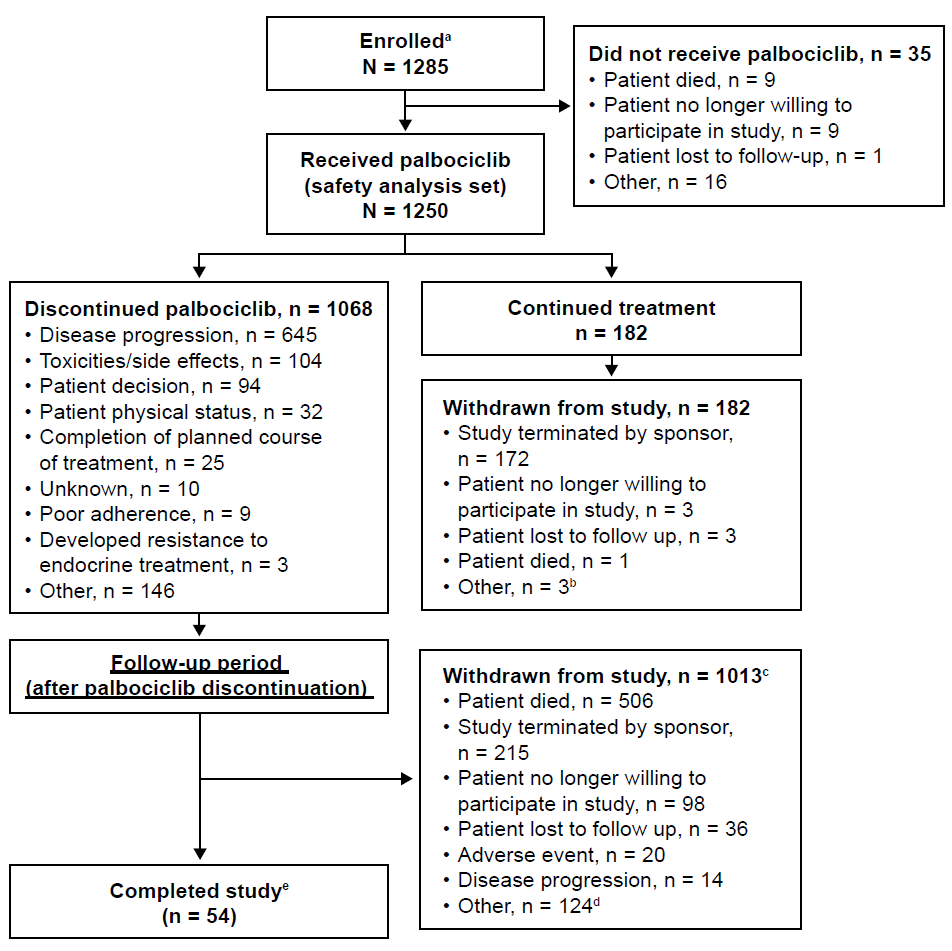
^a^Patient enrollment was between January 04, 2017 and October 03, 2019. The current data cutoff was January 09, 2023.

^b^Other reasons for study withdrawal included patient care transferred to different practice (n = 2) and site decision (n = 1).

^c^1 patient was missing end of study/withdrawal information.

^d^Other reasons for study withdrawal included patient care transferred to different practice or patient moved (n = 84), site no longer participating in study (n = 16), and poor adherence (n = 8), among others (n = 16).

^e^Patients completed 3 years of follow-up after the end of palbociclib treatment.
